# High-Performance Flexible All-Solid-State Supercapacitor from Large Free-Standing Graphene-PEDOT/PSS Films

**DOI:** 10.1038/srep17045

**Published:** 2015-11-20

**Authors:** Yuqing Liu, Bo Weng, Joselito M. Razal, Qun Xu, Chen Zhao, Yuyang Hou, Shayan Seyedin, Rouhollah Jalili, Gordon G. Wallace, Jun Chen

**Affiliations:** 1 ARC Centre of Excellence for Electromaterials Science, Intelligent Polymer Research Institute, Australian Institute of Innovative Materials, Innovation Campus, University of Wollongong, Wollongong, NSW 2522, Australia; 2 Chongqing Key Lab for Advanced Materials & Clean Energies of Technologies, Institute for Clean Energy and Advanced Materials, Southwest University, Beibei, Chongqing, 400715 China; 3 Deakin University, Institute for Frontier Materials, Geelong, VIC 3220, Australia; 4 College of Materials Science and Engineering, Zhengzhou University, Zhengzhou, 450052 China

## Abstract

Although great attention has been paid to wearable electronic devices in recent years, flexible lightweight batteries or supercapacitors with high performance are still not readily available due to the limitations of the flexible electrode inventory. In this work, highly flexible, bendable and conductive rGO-PEDOT/PSS films were prepared using a simple bar-coating method. The assembled device using rGO-PEDOT/PSS electrode could be bent and rolled up without any decrease in electrochemical performance. A relatively high areal capacitance of 448 mF cm^−2^ was achieved at a scan rate of 10 mV s^−1^ using the composite electrode with a high mass loading (8.49 mg cm^−2^), indicating the potential to be used in practical applications. To demonstrate this applicability, a roll-up supercapacitor device was constructed, which illustrated the operation of a green LED light for 20 seconds when fully charged.

The need to develop next-generation wearable and flexible electronics in various fields has promoted the development of highly flexible energy storage devices with high performance. Among various energy storage devices, such as batteries^1^, fuel cells^2^ and supercapacitors (SCs)^2^,^3^,^4^,^5^, SCs are the most promising candidates for flexible devices because of their relatively simple structures, the ease for large scale production, as well as their inherent electrochemical properties (*i.e.* high energy and power density, fast charge-discharge and extremely long cycle life)^3^.

The most crucial factor for fabricating flexible SCs is the development of flexible electrodes with high capacitance and high electrical conductivity to ensure fast charge-discharge. A common method to make flexible electrode is by depositing electroactive materials on soft and flexible substrates with porous structure. For example, carbon dispersions (carbon nanotubes, graphene oxide *etc.*) have been deposited as inks on cellulose papers^6^, porous cotton^7^ and synthetic polymer sponges^8^. Conducting polymers, such as polypyrrole^9^, polyaniline^10^ or poly(3,4-ethylenedioxythiophene)(PEDOT)^11^ have been directly deposited on soft and porous substrates via chemical or electrochemical polymerization. Despite their high flexibility and good ion accessibility, the electrical conductivity of these electrodes has been limited by insulating properties of substrates used affecting the charge-discharge rate of SCs. In addition, the total SC device weight increased due to the use of insulating substrates, leading to a decrease of capacitance per unit weight. To resolve this problem, some researchers have prepared free-standing flexible electrodes using vacuum filtration^12^,^13^,^14^ or spray coating active materials on flexible PET film^15^,^16^,^17^ to create super-thin electrode films. SC devices made from these electrodes often exhibit high gravimetric capacitance. However, the gravimetric capacitance is not maintained when the mass loading or thickness of the films is increased, making them unsuitable for practical applications. The capacitance further decreases when solid-state electrolyte is used because large electrolyte ions could not penetrate into the densely packed electrode materials^14^. In addition, the device flexibility suffers when thicker electrodes are used. Fabrication of highly flexible SC electrode materials with outstanding performance remains a challenge.

Poly(3,4-ethylenedioxythiophene)/poly(styrenesulfonate) (PEDOT/PSS) is considered a promising material for SC electrodes mainly due to its high conductivity, good chemical and electrochemical stability, and excellent dispersibility in various solvents^18^,^19^,^20^. Reduced graphene oxide (rGO) is another promising SC electrode material. It has a high capacitance and an extremely long cycle life due to the ultrahigh specific surface area and the electric double layer mechanism that is the basis of the charge-discharge process. However, the capacitance of pure rGO films is often limited by re-stacking of layers^21^,^22^,^23^,^24^,^25^. Therefore, additives which can prevent re-stacking and simultaneously enhance electrochemical performance have been incorporated into rGOs when used as SC electrodes^21^,^22^,^23^,^24^,^25^.

In this work, we successfully prepared free-standing, large-area and flexible rGO-PEDOT/PSS composite membranes *via* a simple bar-coating method. We demonstrate that these membranes are highly flexible and can be rolled to fabricate SC devices. Commercial PEDOT/PSS was selected as the conductive matrix to prevent re-stacking of rGO layer and at the same time, to impart additional flexibility to the electrodes. The composite films demonstrated high flexibility and conductivity, and when assembled into all-solid-state SCs using PVA-H_3_PO_4_ gel as electrolyte, the devices could be bent at any angle without a significant decrease in electrochemical performance. We demonstrate that the rolled-up SC devices can power a green LED light for 20 seconds when fully charged.

## Results

**Figure 1:**
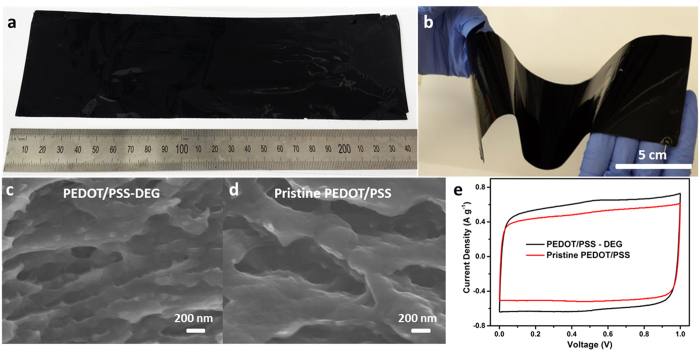
(**a**,**b**) photographs of the as-prepared large size (30 cm × 7 cm), free-standing, and highly flexible PEDOT/PSS film. Cross-sectional SEM images of PEDOT/PSS (**c**) with DEG (Diethylene glycol) film and (**d**) without DEG film. (**e**) Comparison between the specific capacitance of PEDOT/PSS electrodes with and without DEG at 50 mV s^−1^.

A large-size (30 cm × 7 cm) free-standing PEDOT/PSS-DEG film was prepared by bar-coating a thin layer of PEDOT/PSS and diethylene glycol (DEG) solution as described in experimental section. The digital photographs of the free-standing PEDOT/PSS film (14 μm thickness) shown in Fig. 1a,b illustrate that it is highly flexible and can be bent and twisted. Ethylene glycol and DEG are commonly used secondary dopants to improve the conductivity of PEDOT/PSS^18^,^26^,^27^,^28^,^29^,^30^,^31^. A PEDOT/PSS film without DEG (pristine PEDOT/PSS) was also prepared under the same condition as a control. The PEDOT/PSS-DEG has a high electrical conductivity (230 S cm^−1^), which is nearly two orders higher than pristine PEDOT/PSS (3.2 S cm^−1^). This difference in conductivity can be explained to be the result of phase separation of the excess insulating PSS domains from the PEDOT/PSS domains induced by DEG to create a highly conducting PEDOT/PSS network^18^.

A scanning electron microscopy (SEM) study of the cross-sections of the films reveal more macro-scale porosity in PEDOT/PSS-DEG film (Fig. 1d) compared to the pristine PEDOT/PSS film (Fig. 1c). It is assumed that the micro sized pores were previously occupied by DEG when the films were dried at room temperature. Most of the DEG during this step was removed upon oven drying and created macro pores.

The increase in porosity resulted in a better electrochemical performance in terms of specific capacitance (Fig. 1e) because the increase in porosity has increased the accessible surface area. Furthermore, the PEDOT/PSS-DEG film can retain a highly rectangular CV curve when the scan rate was increased to 200 mV s^−1^ (Supplementary Fig. S1), indicative of high charge mobility. The small semi-circle in the high frequency domain of the electrochemical impedance spectra (EIS) also showed low polarisation resistance, indicating fast diffusion of electrolyte ions. These results suggest that the treatment with DEG enhanced the electrical conductivity and porosity of the PEDOT/PSS film significantly as was reflected in the higher specific capacitance. This treatment method was therefore utilized in the preparation of rGO-PEDOT/PSS films and all the samples in the following statement without specific illustration were treated with DEG.

**Figure 2:**
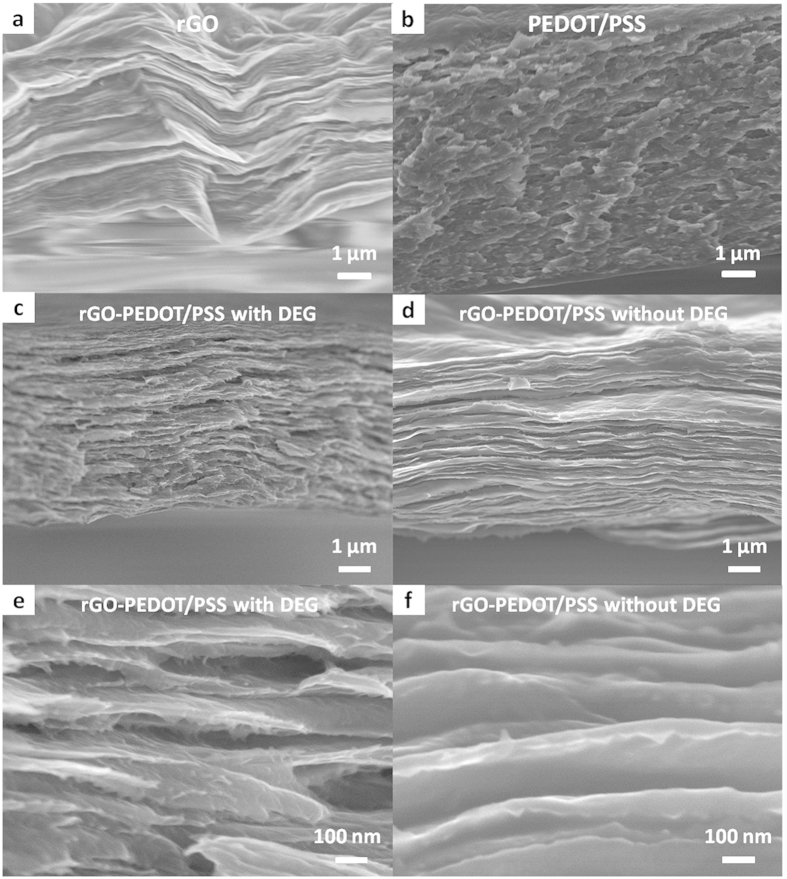
SEM cross-section images of the various films. (**a**) rGO, (**b**) PEDOT/PSS, (**c**) and rGO-PEDOT/PSS with DEG, (**d**) rGO-PEDOT/PSS without DEG, (**e**) rGO-PEDOT/PSS with DEG (higher magnification) and (**f**) rGO-PEDOT/PSS without DEG (higher magnification).

Figure 2 shows the cross-sectional SEM images of rGO, PEDOT/PSS and rGO-PEDOT/PSS composite films. The pristine rGO film (Fig. 2a and Supplementary Fig. S2a) has a layered structure with closed edges, which is formed by re-stacking and interlocking of individual sheets. Such a densely stacked structure has previously been shown to hinder the electrolyte ion diffusion, resulting in an insufficient utilization of rGO’s potential capacitance^13^,^21^,^22^,^32^. When PEDOT/PSS and DEG were incorporated into this system (*i.e.* forming the rGO-PEDOT/PSS film), the cross-section of the film displayed an open edge that could be seen under SEM (Fig. 2c,e, and Supplementary Fig. S2b). This open edge indicates that the presence of PEDOT/PSS and DEG have effectively prevented the re-stacking of individual graphene sheets and provided a higher accessible surface area. In addition, the hydrophilic property of PEDOT/PSS can also help electrolyte ions to penetrate into and access the inner surface of the electrode materials. Images of the composite film without DEG were shown in Fig. 2d,f for comparison. It was found that there is still some re-stacking of several layers of rGO sheets that are separated by PEDOT/PSS. The space between two single layers appears smaller than samples with DEG although it is much larger than pure rGO, showing the addition of DEG in the composite films can also increase the porosity and surface area as in PEDOT/PSS film.

**Figure 3:**
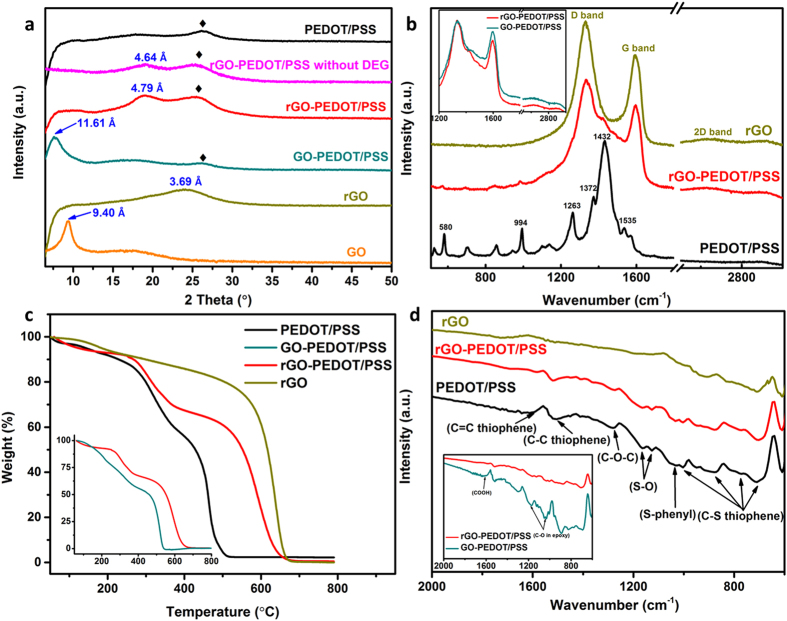
Properties of the rGO, GO, PEDOT/PSS, GO-PEDOT/PSS and rGO-PEDOT/PSS films characterized by (a) XRD, (b) Raman, (c) TGA and (d) FT-IR.

Both pristine and composite materials were also investigated using X-ray diffraction (XRD) measurements. The XRD patterns of PEDOT/PSS, GO, rGO, GO-PEDOT/PSS and rGO-PEDOT/PSS are shown in Fig. 3a. PEDOT/PSS exhibited a peak at 2θ = 25.9°, which is related to the (020) plane of the PEDOT/PSS polymer backbone^33^. This peak is also observed in the XRD spectra of GO-PEDOT/PSS and rGO-PEDOT/PSS. For GO and GO-PEDOT/PSS, the distinct peaks were found at 2θ = 8.8° and 7.6°, respectively. These peaks are correlated to the (002) diffraction of GO sheet, from which the interlayer *d* spacing values have been calculated to be 0.94 nm and 1.16 nm according to Bragg’s law (equation (1), where n = 1, λ is the wavelength of incident wave (1.54 Å))^34^, respectively. This slight increase of interlayer spacing in GO-PEDOT/PSS composite film could be attributed to the good interaction of PEDOT/PSS with individual GO sheets and their intercalation in between GO layers^35^. After reduction by hypophosphorous acid (HPA)^36^, the specific GO peaks at around 10° disappeared in both rGO and rGO-PEDOT/PSS samples and broad peaks were observed at around 2θ = 24.1° for rGO and 2θ = 18.5° for the composite film. These peaks corresponded to interlayer distance that decreased to 0.37 nm and 0.48 nm, respectively. In addition, rGO-PEDOT/PSS composite sample without DEG treatment was also characterized by XRD, with a interlayer distance (0.46 nm) slightly smaller than sample with DEG treatment, further indicating DEG’s role in increasing porosity and surface area.




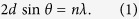




The Raman spectra of samples discussed above are shown in Fig. 3b. The signature peaks for PEDOT/PSS (580, 994, 1263, 1372, 1432 and 1535 cm^−1^) were also found in rGO-PEDOT/PSS composite albeit with significantly weaker intensity^33^. Three main peaks of rGO at 1330, 1590 and 2628 cm^−1^ associated with the D, G and 2D bands, respectively, can also be observed in the composite films. The D/G intensity ratio of the composite films increased from 1.12 (GO-PEDOT/PSS) to 1.25 (rGO-PEDOT/PSS) after HPA treatment suggesting reduction of defects in GO^37^. In addition, the increased 2D intensity because of the recovery of crystallinity also verified the reduction of GO^38^.

The Fourier transform infrared spectra (FTIR) (Fig. 3d) corroborated the Raman results. All of the PEDOT/PSS signature peaks (S-O and S-phenyl bonds in sulfonic acid located at 1167, 1126 and 1029 cm^−1^, respectively; and C = C, C-C and C-S bonds in the thiophene backbone at 1580, 1508, 1001, 894, 771 and 706 cm^−1^, respectively) were observed in the rGO-PEDOT/PSS composite films at lower intensity^33^. From the comparison of GO-PEDOT/PSS and rGO-PEDOT/PSS (shown as inset), the peaks for GO at 1045 cm^−1^ and 1209 cm^−1^ (epoxy C-O stretching vibration) and 1650 cm^−1^ (associated with carboxyl group) can be seen in GO-PEDOT/PSS but not in rGO-PEDOT/PSS, indicating the reduction of GO in the composite films.

The thermal stability of rGO-PEDOT/PSS composites in air was examined by Thermo-gravimetric analysis (TGA). Figure 3c shows the comparison of weight loss of pristine rGO, pristine PEDOT/PSS and rGO-PEDOT/PSS composite. The rGO-PEDOT/PSS composite films show little weight loss below 250 °C indicating a wide operating temperature range, which is important for many applications. Above 250 °C, there was a significant mass loss, which is attributed to the rupture of the sulfonate group from PSS^34^. The steepest weight loss was observed at 500 °C, which is attributed to both the degradation of the polymer backbone and the oxidation of rGO^34^.

The reduction of GO in the composites is also evident in the enhancement of electrical conductivity and electrochemical performance of the samples containing various rGO loading. Supplementary Fig. S3 shows the electrical conductivity of all films before and after reduction. All films containing GO shows a remarkable increase in conductivity after reduction. Among all composite films, the rGO-PEDOT/PSS film with 33 wt.% GO loading displayed the highest conductivity of 92.5 S cm^−1^. In addition, the rGO-PEDOT/PSS film also had the most rectangular shaped CV among the samples investigated, indicating the enhanced double layer capacitance.

**Figure 4:**
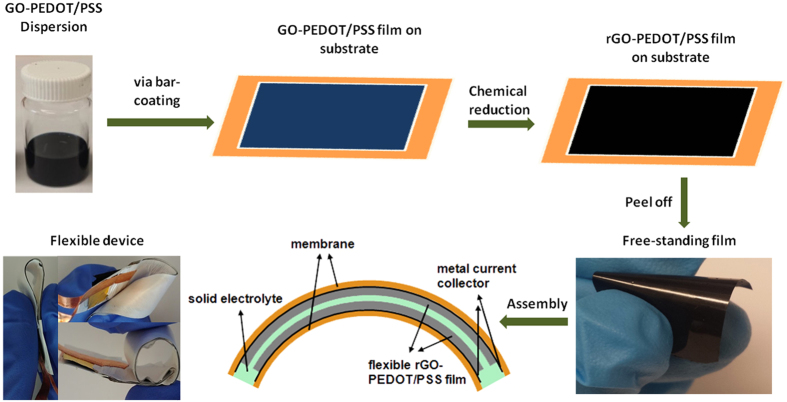
Schematic illustration of the preparation process of rGO-PEDOT/PSS films and the structure of assembled supercapacitor devices.

The rGO-PEDOT/PSS composite films with various GO loadings were prepared and assembled in an all-solid-state supercapacitor (SC) device, as illustrated in Fig. 4. The pristine PEDOT/PSS and pristine rGO films were also prepared using the same method for comparison. The composite films displayed high flexibility and the assembled device could be bent and twisted without impairing the integrity of the device (Fig. 4). Solid-state supercapacitors were fabricated by sandwiching poly(vinyl alcohol) (PVA)/H_3_PO_4_ solid-state electrolyte between two symmetric electrode films. The active area of all fabricated solid-state supercapacitors was set at 2 cm × 0.8 cm. The various mass loadings and thicknesses of the electrode films are presented in Supplementary Table S1. Evaluation of all composite devices revealed that the best electrochemical performance (in terms of capacitance and charge transfer rate) was obtained from the device containing 33 wt.% GO loading (Supplementary Fig. S8), and was therefore selected for further study.

**Figure 5:**
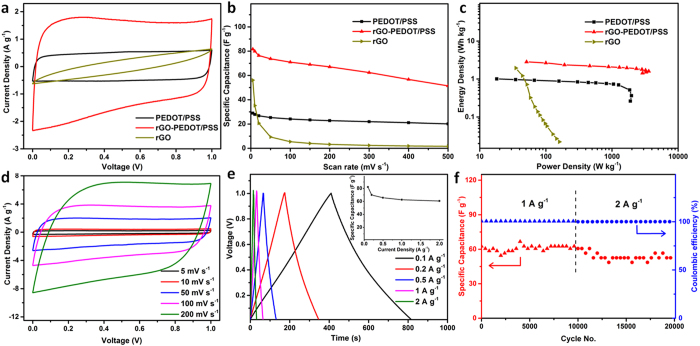
Electrochemical performance of pristine rGO, pristine PEDOT/PSS, and rGO-PEDOT/PSS (33 wt.% GO) SC device. (**a**) CVs of pure rGO, PEDOT/PSS, and rGO-PEDOT/PSS devices at a scan rate of 50 mV s^−1^. (**b**) Specific capacitance of rGO, PEDOT/PSS, and rGO-PEDOT/PSS electrodes calculated from CV. (**c**) Ragone Plot of all the above electrodes. (**d**) CV curves of rGO-PEDOT/PSS at different scan rates (with inset capacitance *vs* current density). (**e**) Galvanostatic charge/discharge curves of rGO-PEDOT/PSS at different current densities. (**f**) Capacitance and Coulombic efficiency of rGO-PEDOT/PSS device during the 20,000 cycles.

The optimal rGO-PEDOT/PSS device (33 wt.% GO) presented superior performance than neat PEDOT/PSS and neat rGO device in terms of capacitance, charge transfer rate, energy and power densities (Fig. 5a–c). The CV curve of this device exhibited a more rectangular shape and larger area than that of the PEDOT/PSS and rGO devices (Fig. 5a), indicating a faster charge transfer rate and a higher capacitance than other samples. Figure 5b shows that the composite device exhibits specific capacitance larger than either of the pure PEDOT/PSS or rGO at all scan rates of 5–500 mV s^−1^. In addition, the decrease in specific capacitance and shape distortion from rectangularity of CV curve of composite film are much lower than pure rGO device when changing from aqueous electrolyte to solid electrolyte (Supplementary Fig. S6). This illustrates that the larger interlayer spacing in the composite films enables the larger ions in the solid electrolyte to penetrate into the interlayer space and access the inner layer surface. Comparison of CV curves and specific capacitance of the composite device with and without DEG (Supplementary Fig. S7) showed enhancement of both capacitance and rate capability of the composite film by the addition of DEG. Figure 5d shows CV curves of rGO-PEDOT/PSS optimal device at different scan rates from 5 mV s^−1^ to 200 mV s^−1^. All CV curves retain their highly rectangular shape, indicating the device’s fast charge transfer rate and good conductivity.

There is also a remarkable improvement in the power and energy densities (based on two electrode weight, calculated from CV data; Supplementary Equation S7 and S8), which can be observed from the Ragone plot in Fig. 5c. The device containing optimal rGO-PEDOT/PSS electrode can achieve a maximum power and energy densities (3,589.5 W kg^−1^ and 2.83 Wh kg^−1^, respectively), which are significantly higher than devices made of pure rGO electrode (159.8 W kg^−1^ and 1.95 Wh kg^−1^, respectively) and pure PEDOT/PSS electrode (1,967.5 W kg^−1^ and 1.00 Wh kg^−1^, respectively). The symmetry of the galvanostatic charge/discharge curves Fig. 5e of the rGO-PEDOT/PSS signifies the excellent capacitive characteristic, with the discharge time decreasing with increasing the applied current, leading to a slight decrease in specific capacitance (inset in Fig. 5e). No significant iR drop is observed at the beginning of the constant current discharge, indicative of the low contact resistance in the device. The long-term charge-discharge performance of this device was also evaluated (Fig. 5f). After 10,000 cycles of constant current charge/discharge at 1 A g^−1^, the capacitance retention was more than 95%. When tested for another 10,000 charge/discharge cycles at a higher current density of 2 A g^−1^, the capacitance remained above 85% of the initial value, suggesting that the device has high stability, long cycle life, and high rate capability. The Coulombic efficiency is also close to 100% for all cycles indicating the high stability of the device (Fig. 5f). This high stability is also shown in the Nyquist plots (Supplementary Fig. S9). A slight decrease in semi-circle of the high frequency region after 10,000 and 20,000 charge/discharge cycles demonstrates decreased polarisation resistance (R_ct_). In addition, the intercept of the real part of impedance with the *x*-axis (R_s_, representative of the resistance of the electrolyte and the contact resistance) also decreased slightly. These results suggest that the inner surface of electrode materials has been fully wetted by the electrolyte after 10,000 cycles with only a minimal mechanical failure from the polymer doping/dedoping and ion absorbing/desorbing process^39^,^40^.

**Figure 6:**
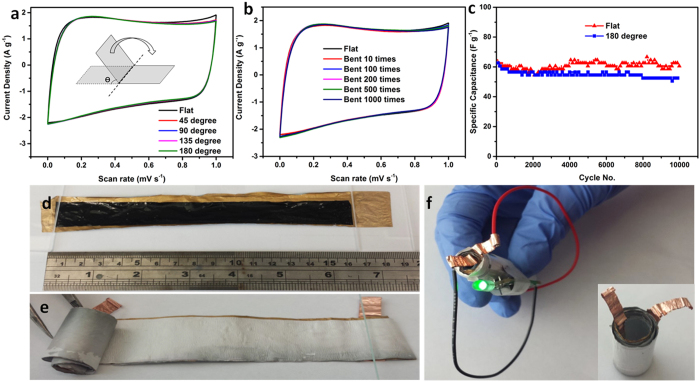
(**a**) CVs of rGO-PEDOT/PSS during bending. Scan rate = 50 mV s^−1^. (**b**) CVs of rGO-PEDOT/PSS after being subject to bending. (**c**) Long-term test of rGO-PEDOT/PSS under flat or 180 degree bended states at a current density of 1 A g^−1^. (**d**) Flexible films coated with solid electrolyte spread out on an Au-coated membrane, (**e**) rolled design and (**f**) the resulting device used to power a green light-emitting diode (LED).

Cyclic voltammetry (CV) (Fig. 6a) and the charge/discharge curves in Supplementary Fig. S9 confirm that no significant change was observed during bending at various angles. Bending up to 1,000 times at 180° did not change the shape of the CV responses (Fig. 6b). The long term galvanostatic tests illustrated that the capacitance tested under 180° after 10,000 cycles only decreased by 11.6% compared with 0°, indicating excellent device performance under large bending angles. In addition, the SC device made from a long strip of electrodes (15 cm × 2 cm) that have been rolled-up as shown in Fig. 6d,e was powerful enough to power a light-emitting diode for 20 seconds when fully charged (Fig. 6f).

## Discussion

We attribute the improved performance (e.g. higher capacitance, better rate capability and energy/power density) of rGO-PEDOT/PSS composite film than pure rGO to the increased porous structure and electrical conductivity. We have now provided higher resolution SEM images that compare the cross-sections of rGO and rGO-PEDOT/PSS films in Supplementary Fig. S2. The pure rGO film shows a densely stacked layer structure while the composite film shows an open edge and more porous structure. In addition, XRD results also showed the supporting evidence that the interlayer distance of rGO has been increased from 0.37 nm (for pure rGO film) to 0.48 nm (for rGO-PEDOT/PSS), confirming the effective intercalation of PEDOT/PSS in between rGO layers (Fig. 3a). These results indicated that the restacking problem of rGO sheets has been effectively prevented in the composite film, resulting in a better utilization of rGO’s high capacitance (189 F g^−1^ for rGO only in composite film than 56.11 F g^−1^ for pure rGO film, supplementary Table S2). Furthermore, the improved conductivity of the composite film (92.5 S cm^−1^) that is over 7 times higher than that of pure rGO film (12.1 S cm^−1^) can explain its more rectangular CV shape and faster charge transfer at high charge-discharge rates.

Notably, the gravimetric capacitance of the optimized rGO-PEDOT/PSS film in this work is only 81 F g^−1^ at the lowest scan rate of 5 mV s^−1^, which is much lower than many literature values of graphene-PEDOT based SCs (100 ~ 300 F g^−1^)^41^,^42^. That is due to the limited gravimetric capacitance (28.9 F g^−1^ even after optimized by DEG) of commercialized PEDOT/PSS utilised in this work. To achieve the aim of industrial large scale fabrication of rGO-PEDOT/PSS large area SCs, commercialised PEDOT/PSS was chosen instead of specially designed high performance PEDOT/PSS^4^ to work as flexible and conductive platform matrix for rGO sheets, which lead to the weaker performance in comparison with previous work. However, the specifically high conductivity of PEDOT-PSS utilised in this work enable the fast charge transfer in the SCs, promising the high mass loading of electrode materials which resulted high areal capacitance.

**Figure 7:**
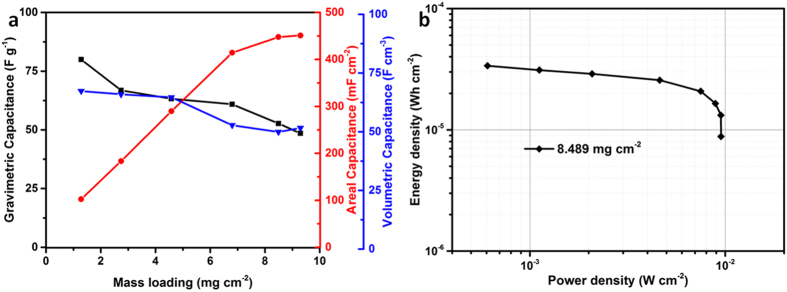
(**a**) Gravimetric, areal and volumetric capacitance *vs*. mass loading at a scan rate of 10 mV s^−1^, (**b**) Ragone plot of rGO-PEDOT/PSS device with a high mass loading of 8.49 mg cm^−2^.

It has been reported that thin electrode films often lead to better specific capacitance performance because electrons and electrolyte ions can be easily transferred^14^,^43^. However, in most practical applications, high mass loading and hence film thickness is necessary to increase device capacity. Here, devices with various electrode material mass loading were fabricated to investigate the relationship between mass loading and specific (*i.e.* areal, volumetric and gravimetric) capacitance of the PEDOT/PSS-rGO composite electrode with 33 wt.% GO loading. As shown in Fig. 7a, the areal capacitance increases with mass loading initially before reaching the highest value of 448 mF cm^−2^ at 8.49 mg cm^−2^ at a scan rate of 10 mV s^−1^. This performance is comparable with previously reported values for graphene (Laser scribing graphene (LSG)^44^, Graphene-Cellulose nanofiber (G-CNF) aerogel^23^, and Graphene-cellulose paper^45^), CNT^14^,^46^ and conducting polymer^10^ solid-state SCs as listed in Supplementary Table S3. The gravimetric capacitance at this loading is 52.7 F g^−1^. Also, the volumetric capacitance (49.9 F cm^−3^) is higher than reported values for solid state graphene (9.6 F cm^−3^)^44^, graphene hydrogel (31 F cm^−3^)^43^ and PEDOT paper (35 F cm^−3^)^47^. In addition, the volumetric capacitance (3.4 F cm^−3^) of the whole SC device (*i.e.* taking into account the mass of the 0.66 mm thick electrode, electrolyte and package membranes) is significantly higher than previous reports CNT (0.30 F cm^−3^)^46^, and graphene (0.42 F cm^−3^)^12^, and comparable with PEDOT-paper (5 F cm^−3^)^47^. The areal capacitances *vs*. mass loading at different scan rates were shown in Supplementary Fig. S10. At a high scan rate of 100 mV s^−1^, the device with 8.49 mg cm^−2^ mass loading can retain an areal capacitance of 300 mF cm^−2^, indicating good rate capability. The symmetric galvanostatic charge/discharge curves (Supplementary Fig. S10) under different current densities signified good capacitive characteristic. The device delivers an energy density of 34 μWh cm^−2^, which is higher than G-CNF aerogel SC (20 μWh cm^−2^)^23^, Graphene hydrogel SC (25.8 μWh cm^−2^)^43^, Graphene cellulose paper SC (2 μWh cm^−2^)^45^ and PEDOT paper SC (17 μWh cm^−2^)^47^. It was also found that the flexibility of the device was not affected by the increase of electrode film thickness. As shown in Fig. S8c and d, the CV responses obtained for these devices have negligible difference when bent at different angles and for 1,000 times at 180°.

In summary, highly flexible free-standing rGO-PEDOT/PSS films have been successfully prepared and fabricated into flexible all-solid-state SC devices using PVA/H_3_PO_4_ as an electrolyte. The incorporation of PEDOT/PSS and DEG into GO sheets increased the interlayer spacing of GO sheets and prevented the GO sheets from re-stacking, which significantly improved the electrochemical performance of the assembled SCs, created more effective surface area and improved the penetration of large sized solid electrolyte ions into the electrode materials. The GO in composite films can be reduced effectively using HPA as the reducing agent, which was verified by FTIR, Raman and XRD and electrical conductivity measurements. The maximum areal capacitance (448 mF cm^−2^) was obtained using 33% rGO-PEDOT/PSS electrode film with 8.49 mg cm^−2^ loading at a scan rate of 10 mV s^−1^. Notably, this composite material performed better than previous reports and showed little changes in capacitance when bent at various angle for 1,000 times. This excellent performance and the ease of fabrication suggest that such electrode materials are good candidates for bendable SC devices that are practical for large scale use.

## Methods

### Materials

Graphite flakes, diethylene glycol (DEG), hydrogen peroxide (H_2_O_2_), poly (vinyl alcohol) (PVA, Mw: 146000 ~ 186000) and hypophosphorous acid (HPA) were purchased from Sigma-Aldrich. Concentrated sulphuric acid (H_2_SO_4_, 98%), orthophosphoric acid (H_3_PO_4_, 85%), and hydrochloric acid (HCl, 32%) were obtained from Chem-Supply. Poly(3,4-ethylenedioxythiophene)/poly(styrenesulfonate) (PEDOT/PSS) pellets was Orgacon™ DRY re-dispersible product from Agfa company.

### Preparation of large PEDOT/PSS flexible films

PEDOT/PSS pellets were dispersed in deionized water at a concentration of 20 mg mL^−1^ by magnetic stirring and then diethylene glycol (DEG) was added to the dispersion at 37.2 mg mL^−1^. The dispersion was stirred overnight and then sonicated (Branson B5500R-DTH bath sonicator, low power) for 30 minutes prior to use. The mixture was bar-coated on a hydrophilic PVDF membrane substrate with glass slides (use side face) encircled to fix films’ shape and size. The thickness of the film was controlled by the volume of dispersion used per unit area of the film. The film was first dried overnight at room temperature and then heated in a 60 °C oven (air atmosphere) overnight to evaporate the remaining water and DEG. A flexible PEDOT/PSS film was then peeled off from the PVDF membrane.

### Preparation of graphene oxide

Graphene Oxide (GO) dispersion was prepared using the modified Hummers method^48^. Firstly, a mixture of concentrated H_2_SO_4_/H_3_PO_4_ (360:40 mL) was added to a mixture of graphite flakes (3.0 g, 1 wt. equiv.) and KMnO_4_ (18.0 g, 6 wt. equiv.). The reaction was heated to 50 °C and stirred for 14.5 hrs, cooled to room temperature, and poured onto ice (~400 mL) with 30% H_2_O_2_ (20 mL). The mixture was then stirred for 30 min and centrifuged at 4,400 rpm for 20 min. The precipitate was washed and centrifuged with HCl solution (9:1 water/HCl by volume) twice and then dispersed in water and dialyzed for 7 days. The graphene oxide dispersion was finally obtained by probe sonicating (Branson Digital sonifier, 400 watt, 38% amplitude) the purified graphite oxide dispersion for 1 hr, with a pulse of two seconds on and one second off, totally 1.5 hrs.

### Preparation of rGO-PEDOT/PSS flexible films

The composite dispersions were prepared by the addition of PEDOT/PSS into the GO dispersions. Samples with various GO loadings of 20 wt.%, 33 wt.%, 50 wt.%, 67 wt.% and 80 wt.% were prepared. In each sample, the total concentration (solid content) of PEDOT/PSS and GO was kept at 20 mg mL^−1^. Diethylene glycol was added to each sample at 33.2 mg/mL^−1^. GO-PEDOT/PSS films were prepared the same way as PEDOT/PSS films. All films were immersed in 5 wt.% HPA and then heated to 60 °C for 24 hours, rinsed with water and dried at room temperature.

### Electrochemical characterization

Films were assembled into two electrode all-solid-state symmetric supercapacitor devices by the following methods. A H_3_PO_4_/PVA gel electrolyte was prepared by mixing PVA powder (4 g), H_3_PO_4_ (6 g) and deionized water (40 mL) together. The mixture was heated to around 85 °C under magnetic stirring until the solution became clear. 150 nm gold was sputter coated directly on one side of electrode film which served as current collector. Hot PVA/H_3_PO_4_ electrolyte (heated to 85 °C prior to use) was drop cast onto the other side of the electrode films. Films were left in air overnight to evaporate most of the water contained in electrolyte. Two films were then pressed together (with both of the electrolyte side pressed face-to-face) to form an all-solid-state flexible symmetric supercapacitors.

Cyclic voltammetry (CV) test of the assembled devices were performed using a CHI720 electrochemical work station. Galvanostatic cycling tests were carried out with a Neware Galvanostat (100 mA, 5 V) equipped with Test Control V.5.0 software. All calculations of capacitances, energy and power densities are according to Supplementary Equation S2-S8. The potential window studied was between 0.01 V to 1 V. Electrochemical impedance spectroscopy (EIS) were obtained using Solartron SI1260 Impedance Analyser and EG&G Instruments Princeton Applied Research Potentiostat/Galvanostat Model 283, and employing a frequency range of 100 kHz to 0.01 Hz and an AC amplitude of 10 mV at open circuit potential.

### Physical characterization

Scanning electron microscopy (SEM) images were obtained from a JEOL JSM-7500FA field emission SEM in which the accelerating voltage was set at 5.0 kV and the emission current was 10 mA. X-ray diffraction (XRD) was performed on a GBC MMA XRD (λ = 1.54 Å) with the voltage and current kept at −40 kV and 25 mA, respectively. Thermo-gravimetric analysis (TGA) was carried out in air using Q500 (TA Instruments) with data analysis carried out using the Q Series software V. 2.5.0.255. The temperature range studied is between 50 °C to 800 °C at rate of 5 °C/min. Fourier transform infrared spectroscopy (FT-IR) was performed using the Shimadzu AIM8000 FT-IR spectrometer. Raman spectroscopy was carried out on a Jobin-Yvon Horbia 800 using a 632.81 nm laser. The data analysis was carried out using Labspec V.5.45.09 software.

## Additional Information

**How to cite this article**: Liu, Y. *et al.* High-Performance Flexible All-Solid-State Supercapacitor from Large Free-Standing Graphene-PEDOT/PSS Films. *Sci. Rep.* **5**, 17045; doi: 10.1038/srep17045 (2015).

## Supplementary Material

Supplementary Information

## References

[b1] Yoshio M., Brodd R. J. & Kozawa A. Lithium-ion batteries: science and technologies. Springer (2009).

[b2] Winter M. & Brodd R. J. What are batteries, fuel cells and supercapacitors? Chem. Rev. 104, 4245–4269 (2004). 15669155 10.1021/cr020730k

[b3] Miller J. R. & Simon P. Electrochemical capacitors for energy management. Science (New York, NY) 321, 651–652 (2008). 10.1126/science.1158736 18669852

[b4] Pech D. *et al.* Ultrahigh-power micrometre-sized supercapacitors based on onion-like carbon. Nat. Nanotechnol. 5, 651–654 (2010). 20711179 10.1038/nnano.2010.162

[b5] Lang X., Hirata A., Fujita T. & Chen M. Nanoporous metal/oxide hybrid electrodes for electrochemical supercapacitors. Nat. Nanotechnol. 6, 232–236 (2011). 21336267 10.1038/nnano.2011.13

[b6] Kang Y. J., Chung H., Han C.-H. & Kim W. All-solid-state flexible supercapacitors based on papers coated with carbon nanotubes and ionic-liquid-based gel electrolytes. Nanotechnology 23, 289501 (2012). 10.1088/0957-4484/23/6/065401 22248712

[b7] Hu L. *et al.* Stretchable, porous, and conductive energy textiles. Nano Lett. 10, 708–714 (2010). 20050691 10.1021/nl903949m

[b8] Chen W. *et al.* High-performance nanostructured supercapacitors on a sponge. Nano Lett. 11, 5165–5172 (2011). 21923166 10.1021/nl2023433

[b9] Yuan L. *et al.* Polypyrrole-coated paper for flexible solid-state energy storage. Energy Environ. Sci. 6, 470–476 (2013).

[b10] Yuan L. *et al.* Paper-based supercapacitors for self-powered nanosystems. Angew. Chem. Int. Ed. 51, 4934–4938 (2012). 10.1002/anie.201109142 22473807

[b11] Anothumakkool B., Torris, A. T. A., Bhange S. N., Badiger M. V. & Kurungot S. Electrodeposited polyethylenedioxythiophene with infiltrated gel electrolyte interface: a close contest of an all-solid-state supercapacitor with its liquid-state counterpart. Nanoscale 6, 5944–5952 (2014). 24764081 10.1039/c4nr00659c

[b12] Moon I. K., Lee J., Ruoff R. S. & Lee H. Reduced graphene oxide by chemical graphitization. Nat. Commun. 1, 73 (2010). 20865806 10.1038/ncomms1067

[b13] Choi B. G., Hong J., Hong W. H., Hammond P. T. & Park H. Facilitated Ion Transport in all-solid-state flexible supercapacitors. ACS Nano 5(9), 7205–7213 (2011). 21823578 10.1021/nn202020w

[b14] Jo K. *et al.* Stable aqueous dispersion of reduced graphene nanosheets via non-covalent functionalization with conducting polymers and application in transparent electrodes. Langmuir 27, 2014–2018 (2011). 21226499 10.1021/la104420p

[b15] Kaempgen M., Chan C. K., Ma J., Cui Y. & Gruner G. Printable thin film supercapacitors using single-walled carbon nanotubes. Nano lett. 9, 1872–1876 (2009). 19348455 10.1021/nl8038579

[b16] Wu Z. S., Liu Z., Parvez K., Feng X. & Mullen K. Ultrathin Printable Graphene Supercapacitors with AC Line-Filtering Performance. Adv. Mater. 27, 3669–3675 (2015). 25973974 10.1002/adma.201501208

[b17] Choi K. S., Liu F., Choi J. S. & Seo T. S. Fabrication of free-standing multilayered graphene and poly(3,4-ethylenedioxythiophene) composite films with enhanced conductive and mechanical properties. Langmuir 26, 12902–12908 (2010). 20617852 10.1021/la101698j

[b18] Crispin X. *et al.* The origin of the high conductivity of (PEDOT - PSS) plastic electrodes. Chem. Mater. 18, 4354–4360 (2006).

[b19] Ryu K. S. *et al.* Poly(ethylenedioxythiophene) (PEDOT) as polymer electrode in redox supercapacitor. Electrochim. Acta 50, 843–847 (2004).

[b20] Snook G. A., Kao P. & Best A. S. Conducting-polymer-based supercapacitor devices and electrodes. J. Power Sources 196, 1–12 (2011).

[b21] Wu Q., Xu Y., Yao Z., Liu A. & Shi G. Supercapacitors based on flexible graphene/polyaniline nanofiber composite films. ACS Nano 4(4), 1963–1970 (2010). 20355733 10.1021/nn1000035

[b22] Li Z. *et al.* Flexible graphene/MnO_2_ composite papers for supercapacitor electrodes. J. Mater. Chem. 21, 14706–14706 (2011).

[b23] Gao K. *et al.* Cellulose nanofiber–graphene all solid-state flexible supercapacitors. J. Mater. Chem. A 1, 63–63 (2013).

[b24] Sun Y. & Shi G. Graphene/polymer composites for energy applications. J. Polym. Sci. Part B Polym. Phys. 51, 231–253 (2013).

[b25] Yan J., Wang Q., Wei T. & Fan Z. Recent Advances in Design and Fabrication of Electrochemical Supercapacitors with High Energy Densities. Adv. Energy Mater. 4, 1300816 (2014).

[b26] Kim J. Y., Jung J. H., Lee D. E. & Joo J. Enhancement of electrical conductivity by a change of solvents. Synt. Met. 126, 311–316 (2002).

[b27] Crispin X. *et al.* Stability of Poly (3, 4-ethylene dioxythiophene)– Poly (styrene sulfonate): A Photoelectron Spectroscopy Study. J. Polym. Sci. Part B Polym. Phys. 41, 2561–2583 (2003).

[b28] Jalili R., Razal J. M., Innis P. C. & Wallace G. G. One-Step Wet-Spinning Process of Poly(3,4-ethylenedioxythiophene):Poly(styrenesulfonate) Fibers and the Origin of Higher Electrical Conductivity. Adv. Funct. Mater. 21, 3363–3370 (2011).

[b29] Ouyang J., Chu C. W., Chen F. C., Xu Q. & Yang Y. High-Conductivity Poly(3,4-ethylenedioxythiophene):Poly(styrene sulfonate) Film and Its Application in Polymer Optoelectronic Devices. Adv. Funct. Mater. 15, 203–208 (2005).

[b30] Jalili R., Razal J. M. & Wallace G. G. Exploiting high quality PEDOT:PSS–SWNT composite formulations for wet-spinning multifunctional fibers. J. Mater. Chem. 22, 25174–25182 (2012).

[b31] Jalili R., Razal J. M. & Wallace G. G. Wet-spinning of PEDOT:PSS/functionalized-SWNTs composite: a facile route toward production of strong and highly conducting multifunctional fibers. Sci. Rep. 3, 3438 (2013). 24336593 10.1038/srep03438 PMC3863815

[b32] Wang D. W. *et al.* Fabrication of Graphene/Polyaniline composite paper via *in situ* anodic electropolymerization for high-performance flexible electrode. ACS Nano 3(7), 1745–1752 (2009). 19489559 10.1021/nn900297m

[b33] Zhang X., Chang D., Liu J. & Luo Y. Conducting polymer aerogels from supercritical CO_2_ drying PEDOT-PSS hydrogels. J. Mater. Chem. 20, 5080–5080 (2010).

[b34] Antiohos D. *et al.* Performance enhancement of single-walled nanotube–microwave exfoliated graphene oxide composite electrodes using a stacked electrode configuration. J. Mater. Chem. A 2, 14835–14835 (2014).

[b35] Zhang K., Zhang L. L., Zhao X. S. & Wu J. Graphene/Polyaniline nanofiber composites as supercapacitor electrodes. Chem. Mater. 22, 1392–1401 (2010).

[b36] Wang X. *et al.* A facile and cost-effective approach to the reduction of exfoliated graphite oxide using sodium hypophosphite under acidic conditions. J. Mater. Chem. C 1, 690–694 (2013).

[b37] Stankovich S. *et al.* Synthesis of graphene-based nanosheets via chemical reduction of exfoliated graphite oxide. Carbon 45, 1558–1565 (2007).

[b38] Chen J. *et al.* Scalable solid-template reduction for designed reduced graphene oxide architectures. ACS Appl. Mater. Interfaces 5, 7676–7681 (2013). 23790146 10.1021/am402084y

[b39] Antiohos D. *et al.* Manganosite–microwave exfoliated graphene oxide composites for asymmetric supercapacitor device applications. Electrochim. Acta 101, 99–108 (2013).

[b40] Antiohos D. *et al.* Compositional effects of PEDOT-PSS/single walled carbon nanotube films on supercapacitor device performance. J. Mater. Chem. 21, 15987–15994 (2011).

[b41] Alvi F. *et al.* Graphene–polyethylenedioxythiophene conducting polymer nanocomposite based supercapacitor. Electrochim. Acta 56, 9406–9412 (2011).

[b42] Zhang J. & Zhao X. S. Conducting polymers directly coated on reduced graphene oxide sheets as high-performance supercapacitor electrodes. J. Phys. Chem. C 116, 5420–5426 (2012)

[b43] Xu Y. *et al.* Flexible solid-state supercapacitors based on three-dimensional. ACS Nano 7(5), 4042–4049 (2013). 23550832 10.1021/nn4000836

[b44] El-Kady M. F., Strong V., Dubin S. & Kaner R. B. Laser scribing of high-performance and flexible graphene-based electrochemical capacitors. Science (New York, NY) 335, 1326–1330 (2012). 10.1126/science.1216744 22422977

[b45] Weng Z. *et al.* Graphene-cellulose paper flexible supercapacitors. Adv. Energy Mater. 1, 917–922 (2011).

[b46] Kang Y. J. *et al.* All-solid-state flexible supercapacitors fabricated with bacterial nanocellulose papers, carbon nanotubes, and triblock-copolymer ion gels. ACS Nano 6(7), 6400–6406 (2012). 22717174 10.1021/nn301971r

[b47] Anothumakkool B., Bhange S. N., Soni R. & Kurungot S. Novel scalable synthesis of highly conducting and robust PEDOT paper for high performance flexible solid-supercapacitor. Energy Environ. Sci. 8, 1339–1347 (2015).

[b48] Marcano D. C. *et al.* Improved synthesis of graphene oxide. ACS Nano 4(8), 4806–4814 (2010). 20731455 10.1021/nn1006368

