# Supplementary material for: High-Performance Flexible All-Solid-State Supercapacitor from Large Free-Standing Graphene-PEDOT/PSS Films
Source: Sci Rep. 2015 Nov 20;5:17045. doi: 10.1038/srep17045 (PMC4653634; doi:10.1038/srep17045)
Supplement: Supplementary Information [file srep17045-s1.doc]

**Supplementary Information**

**High-Performance Flexible All-Solid-State Supercapacitor from Large Free-Standing Graphene-PEDOT/PSS Films**

Yuqing Liu^Ɨ^, Bo Weng^Ɨ^, Joselito M. Razal^*^, Qun Xu, Chen Zhao, Yuyang Hou, Shayan Seyedin, Rouhollah Jalili, Gordon G. Wallace, Jun Chen^*^

1. **Performance of PEDOT/PSS-DEG supercapacitor device**


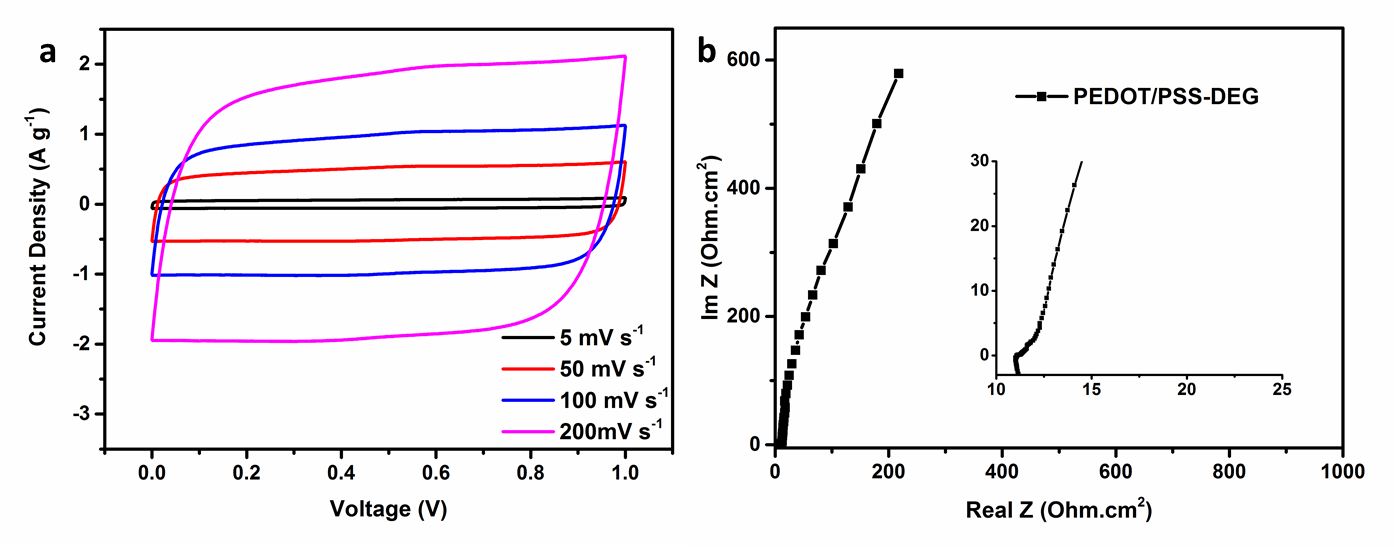


**Figure S1** | (a) Cyclic voltammetry (CV) curves of PEDOT/PSS-DEG supercapacitor device at different scan rates. (b) Electrochemical impedance spectrum (EIS) of PEDOT/PSS-DEG device.

1. **SEM images of rGO-PEDOT/PSS with and without DEG (higher magnification)**


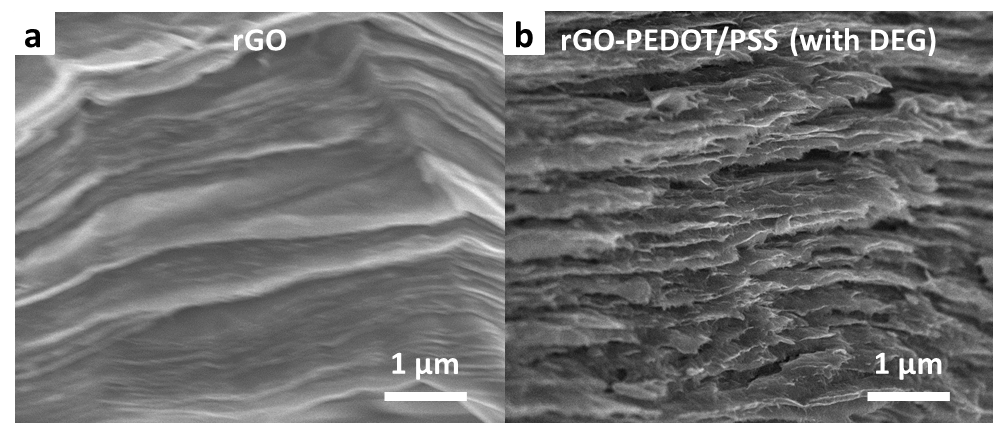


**Figure S2** | Higher magnification SEM cross-section images of rGO-PEDOT/PSS composite films (a) with DEG and (b) without DEG

1. **Effect of HPA treatment on GO, PEDOT/PSS and GO-PEDOT/PSS**


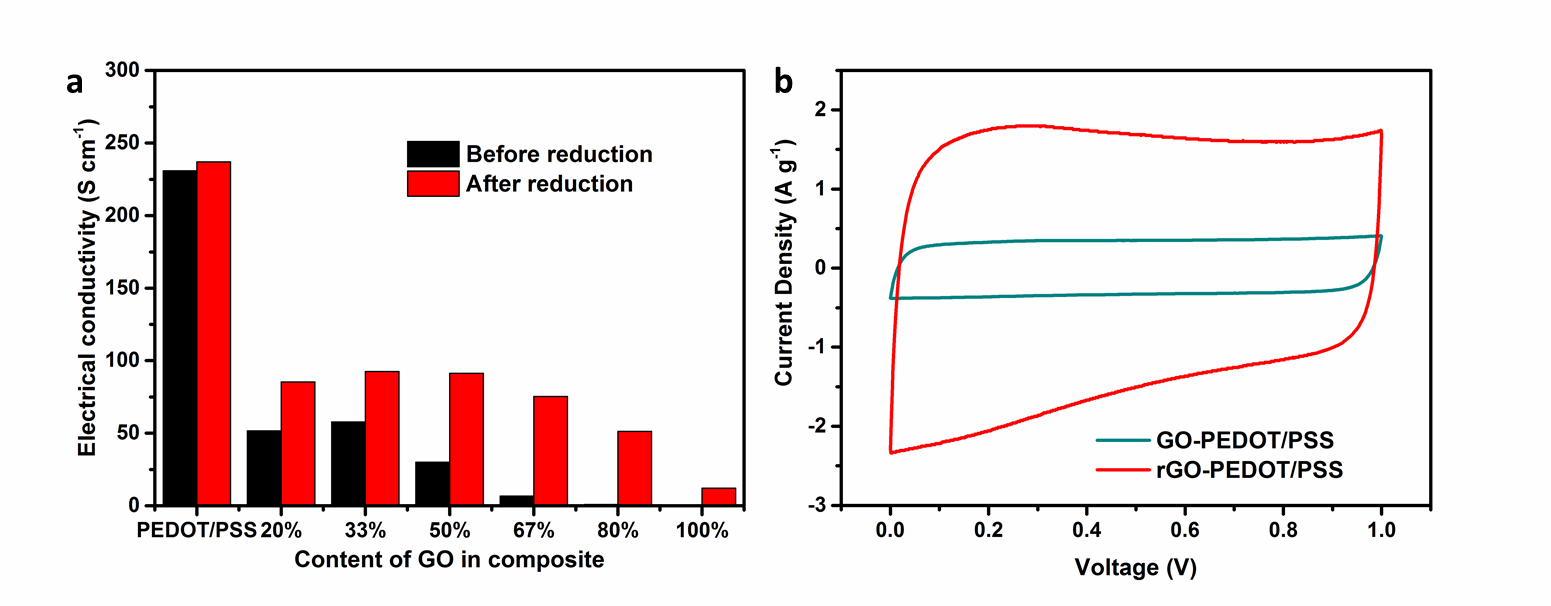


**Figure S3** | (a) Electrical conductivity of different films before and after HPA treatment. (b) CV curves of GO-PEDOT/PSS composite device with 33 wt. % GO before and after HPA treatment (scan rate=50 mV s^-1^).


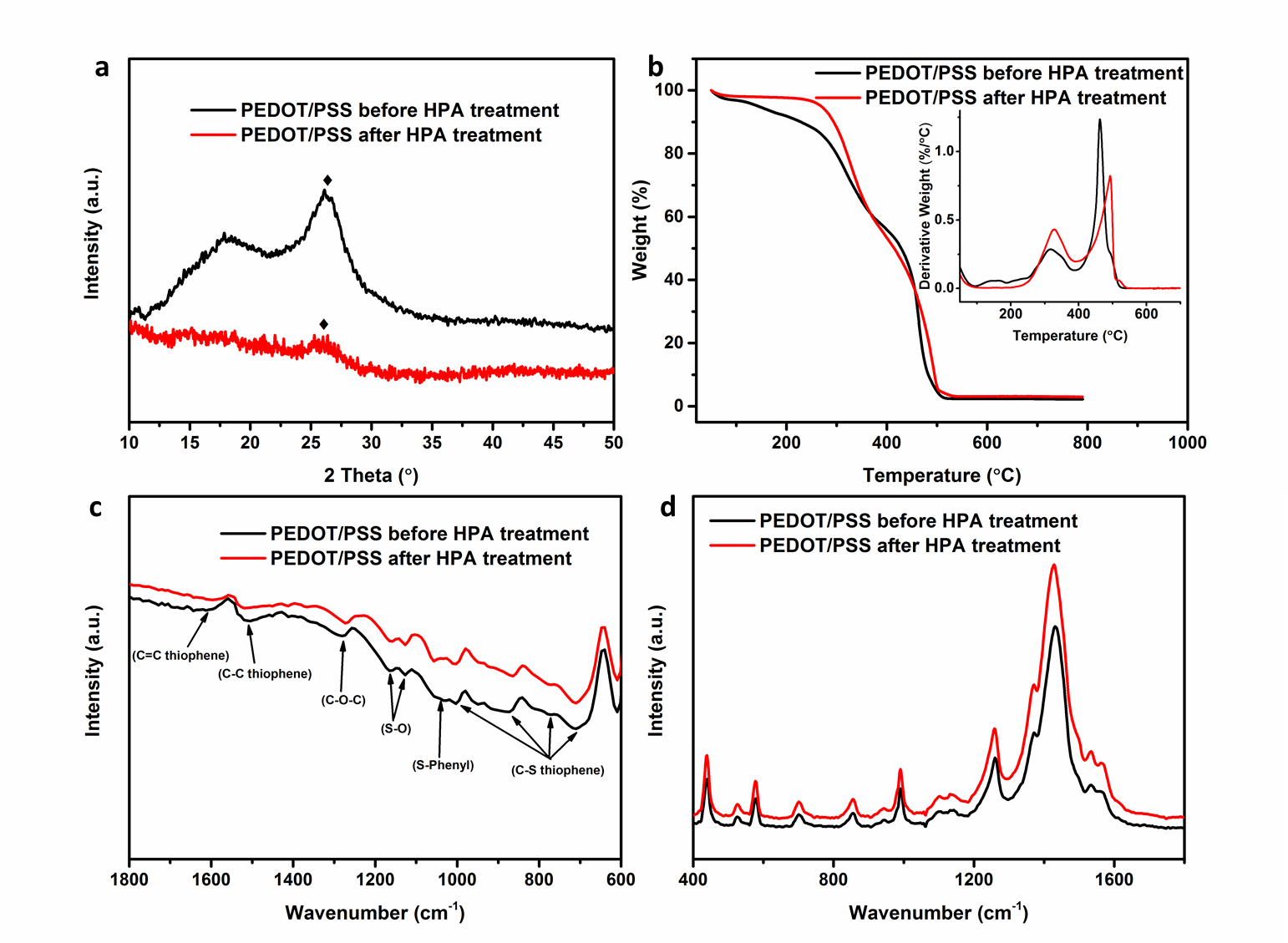


**Figure S4 | Physical characterization of PEDOT/PSS before and after HPA treatment.** (a) XRD, (b) TGA, (c) FT-IR and (d) Raman spectra.

To ensure that PEDOT/PSS was not damaged by HPA when the composite films were treated using HPA, we characterized the films before and after HPA treatment using a four-probe conductivity, FT-IR, Raman, XRD and TGA. Electrical conductivity of PEDOT/PSS film had a negligible increase (Figure S2a) with HPA treatment and the main peak at 2θ = 25.9⁰ for PEDOT/PSS in XRD can still be observed as shown in Figure S4a after HPA reduction. In FTIR and Raman, all the signature peaks PEDOT/PSS remained the same after HPA reduction (Figure S4c and d). In addition, the steepest degradation peak of PEDOT/PSS before and after HPA occurred at 500 °C due to polymer backbone rupturing (Figure S4b), which also indicates that the polymer backbone was not damaged by HPA.


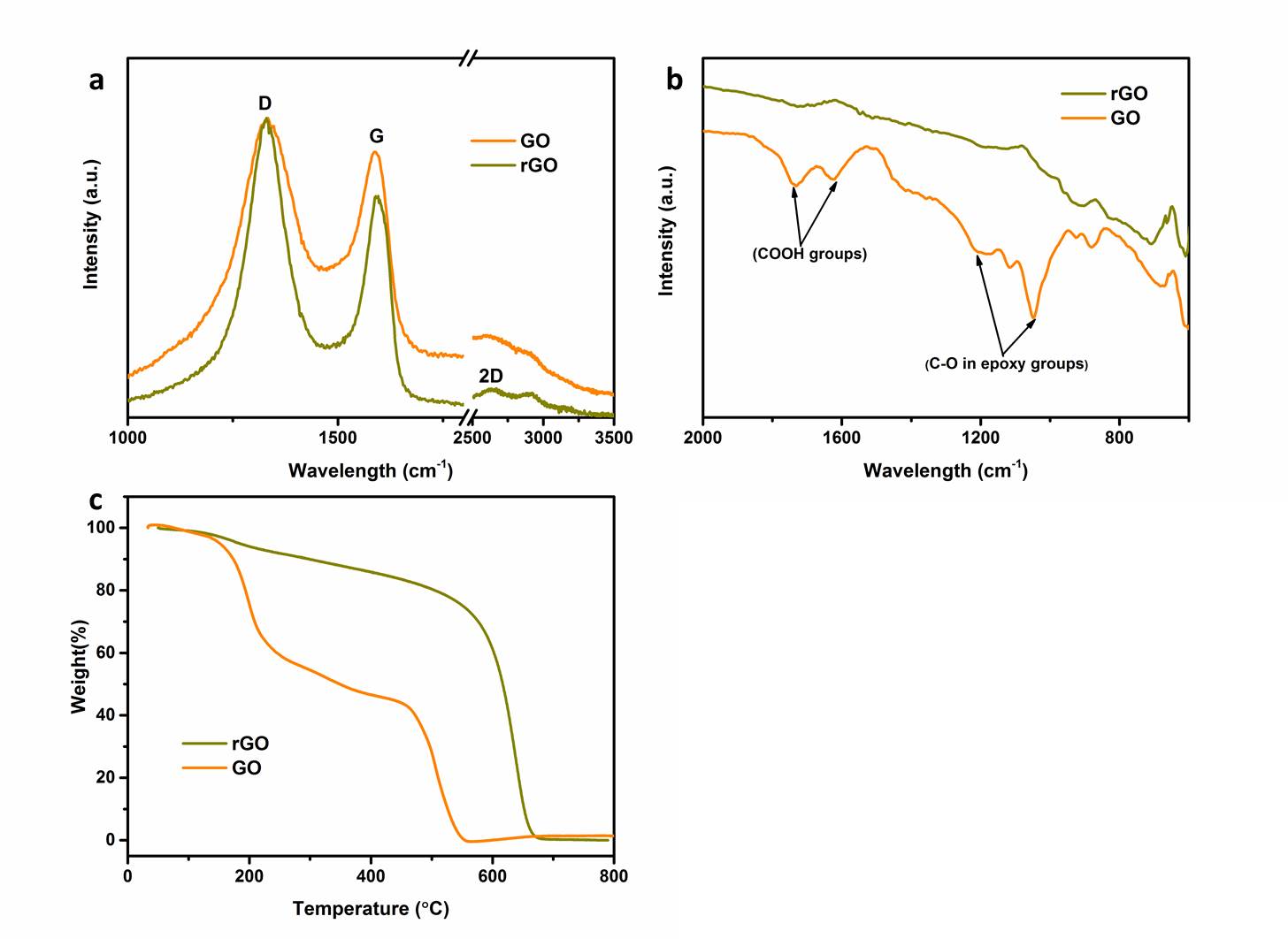


**Figure S5 | Physical characterization of GO and rGO.** (a) Raman, (b) FT-IR and (c) TGA

Pure graphene oxide film was also confirmed to be reduced by HPA using Raman, FT-IR and TGA characterizations as shown in Figure S5. After HPA treatment, the D and G bands (at 1330 and 1590 cm^-1^ respectively) were still present, but the value of I_D_/I_G_ intensity ratio increased compared with that of the GO film. This change suggests that GO was chemically converted to rGO after HPA treatment according to Rough et al^1^. The increased 2D intensity due to the recovery of crystallinity also verified the formation of rGO^2^. After reduction, the peak intensity of C-O stretching (at 1045 and 1209 cm^-1^) and COOH stretching (at 1624 and 1732 cm^-1^) in FT-IR spectra decreased greatly, indicating that most GO sheets in the films were reduced^3^. As shown in Figure S5c, GO is thermally unstable and starts to loose mass at 200 °C due to the pyrolysis of labile oxygen-containing functional groups and the burn of functional groups resulted in an advanced carbon pyrolysis at 500 °C, which occurred from 600 °C for reduced graphene oxide. This indicates the removal of thermally labile oxygen functional groups during reduction process^1^.

1. **Performance comparison in different electrolytes**


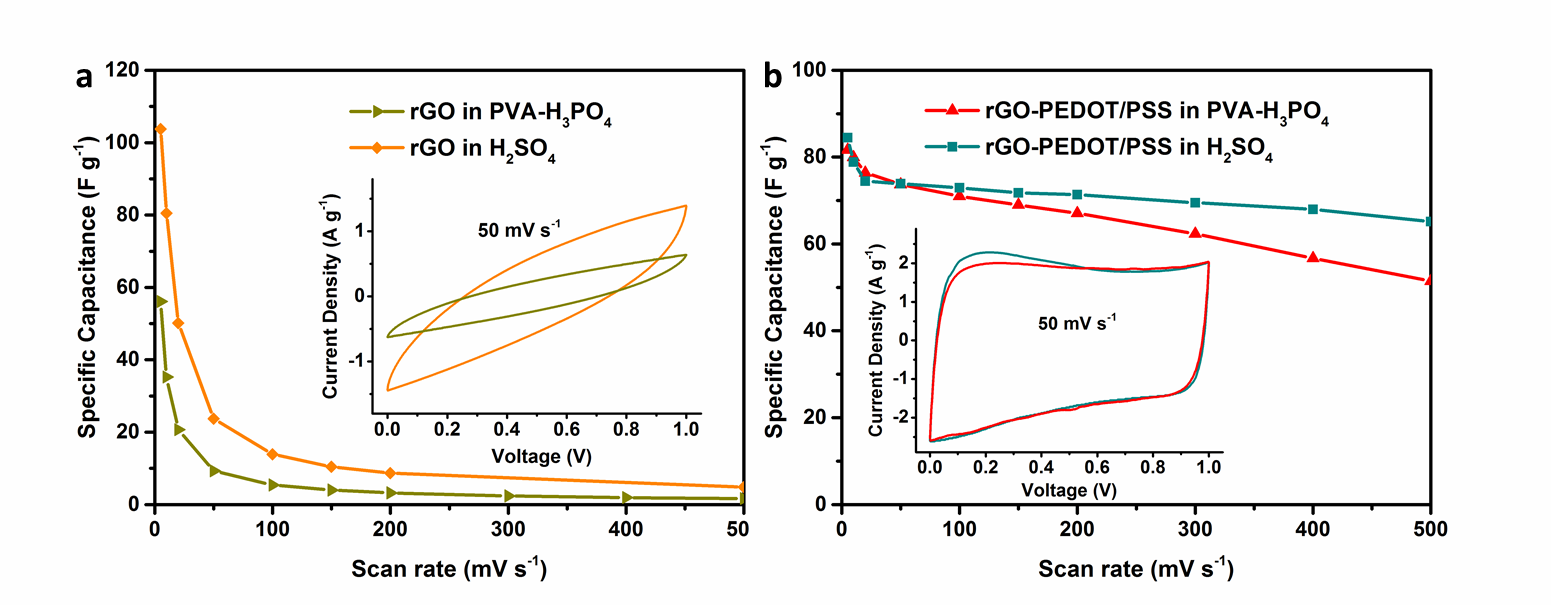


**Figure S6** | Performance comparison of (a) rGO and (b) rGO-PEDOT/PSS (33 wt. % GO) in aqueous and solid electrolyte.

1. **Performance comparison of rGO-PEDOT/PSS composite device with and without DEG**


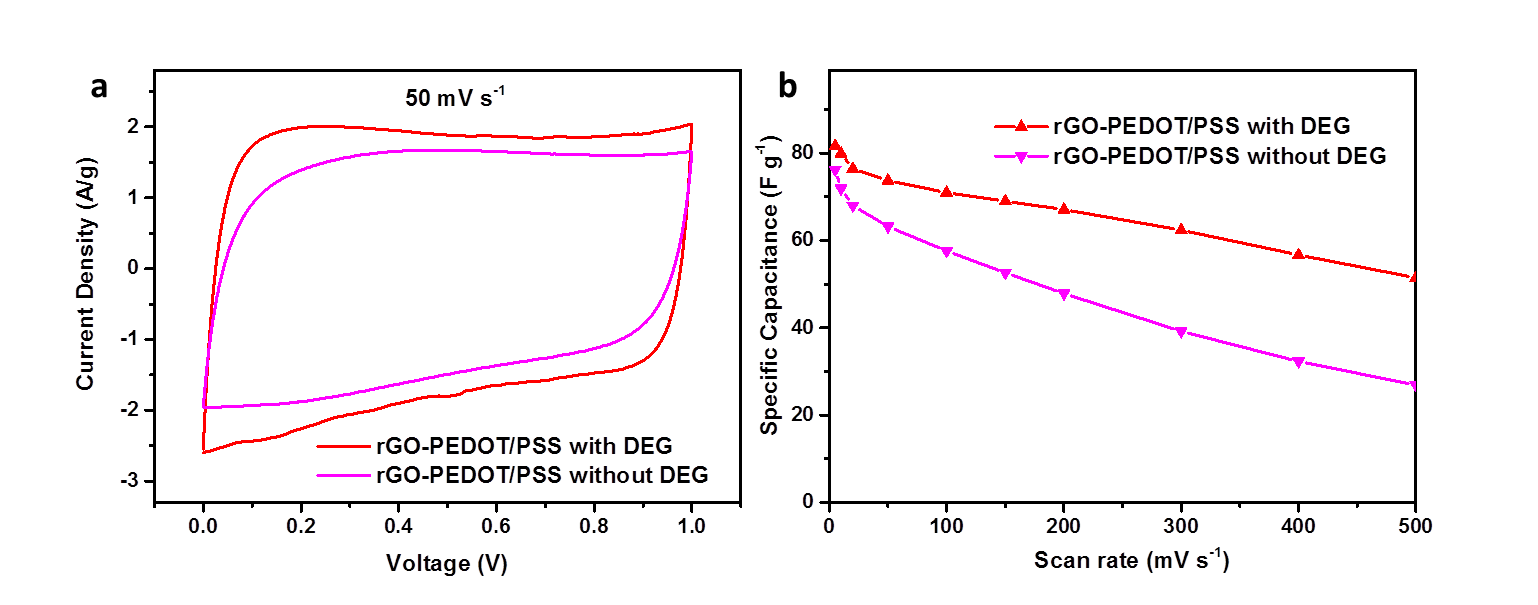


**Figure S7 | Performance comparison of rGO-PEDOT/PSS (33 wt. % GO) with DEG and without DEG.** (a) CV curves at a scan rate of 50 mV s^-1^, (b) Specific capacitance *vs* scan rate.

1. **Electrochemical performance of pure rGO, PEDOT/PSS and rGO-PEDOT/PSS with different composite ratio**

Figure S8 compares the electrochemical performance of pure PEDOT/PSS, pure rGO, and rGO-PEDOT/PSS composite devices. In Figure S8a, the CV curves of all devices at a low scan rate of 5 mV s^-1^ show that all devices except for rGO device have rectangular shape. When the scan rate increased to 200 mV s^-1^, only pure PEDOT/PSS, 20 wt. % GO and 33 wt. % GO loading composite device maintained their shape and 33 wt. % GO had the highest current density. This is because rGO has the lowest conductivity as shown in Figure S8a, which leads to a slow charge-discharge response. The specific capacitances of all electrodes calculated from the CV curves were shown in figure S8c. The addition of rGO significantly improved the specific capacitance of PEDOT/PSS electrodes, from 25 F g^-1^ for PEDOT/PSS to 112 F g^-1^ for 80 wt. % GO electrode. However, for pure rGO electrodes, the capacitance is significantly lower than all composites. This is attributed to the re-stacking of rGO. The gel electrolyte is not able to penetrate fully into the inner layer of rGO sheets and ions cannot access the inner surface, leading to insufficient utilization of the high capacitance inherent to rGO. In addition, the specific capacitance of all electrode materials decreased with increasing scan rates. Films with 20 wt. % and 33 wt. % GO loading showed that capacitance decreased much slower than the other rGO loadings. Although electrodes with 20 wt. % and 33 wt. % GO loadings have relatively low capacitance of 70 F g^-1^ and 81 F g^-1^, respectively, they can retain much higher capacitance (50 F g^-1^) than any ratios at high scan rate of 500 mV s^-1^, which is better than literature values reported for other carbon materials. As 33 wt. % loading device has a higher capacitance than the 20 wt. % loading, 33 wt. % is considered to be the best GO loading for rGO-PEDOT/PSS supercapacitor devices. Areal capacitance and volumetric capacitance were also calculated and listed in Table S1. It has shown that the highest areal capacitance at scan rates of 50 mV s^-1^ and 200 mV s^-1^ were also achieved by 33 wt. % GO loading device. Despite that 33 wt. % device has a slight smaller volumetric capacitance (55 F cm^-3^) than the 20 wt. % device (56.5 F cm^-3^) due to the higher thickness (leading to smaller density), it is still considered as the best loading when considering all the capacitance in terms of gravimetric, areal and volume. The nyquist plots in Figure S8d are characteristic of capacitive type behaviour. In the low frequency region of the spectrum, all the plots except for rGO tend towards a vertical line where the imaginary part of impedance rapidly increases, which is characteristic of capacitive behaviour. The width of the high frequency semi-circle in nyquist plot corresponds to the polarisation resistance (R_p_). We can see from figure S8d that R_p_ increased with the addition of rGO, showing that the existence of rGO has adverse effects on the rate of electrolyte diffusion.


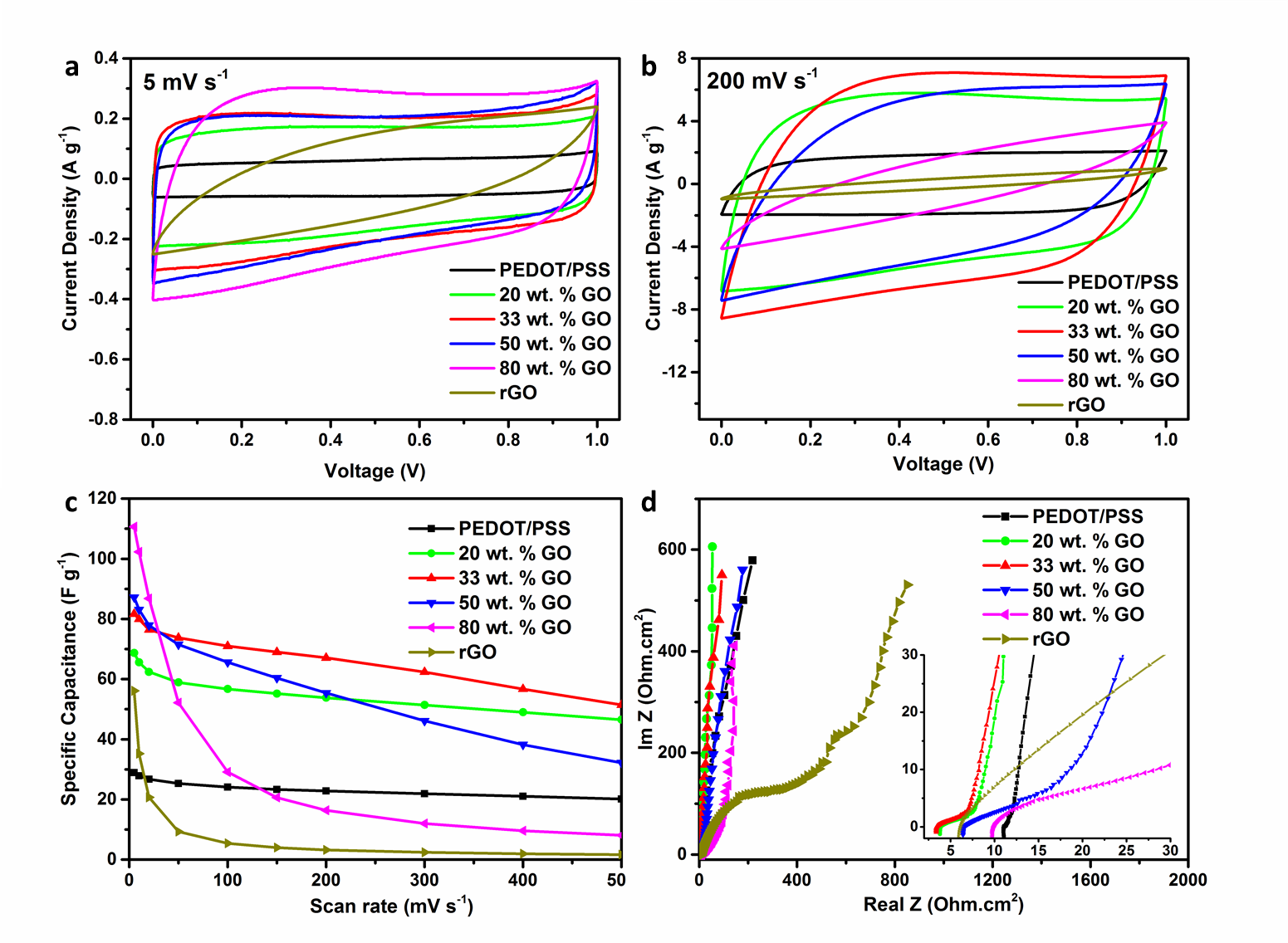


**Figure S8 | Electrochemical properties of pristine PEDOT/PSS, pristine rGO and rGO-PEDOT/PSS composites (with different GO loadings) devices**. (a) CV curves at a can rate of 5 mV s^-1^. (b) CV curves at a scan rate of 200 mV s^-1^. (c) Specific capacitance vs scan rate of the electrodes. (d) Nyquist plots.

**Table S1 Mass loading and thickness of pure PEDOT/PSS film, pure rGO film and rGO-PEDOT/PSS films with different GO loadings**

| **Different films** | | **PEDOT/PSS** | **rGO-PEDOT/PSS with different GO loadings** | | | | | **rGO** |
| --- | --- | --- | --- | --- | --- | --- | --- | --- |
|  |  |  | **20%** | **33%** | **50%** | **67%** | **80%** |  |
| **Mass loading (mg cm^-2^)** | | 2.00 | 1.22 | 1.28 | 1.12 | 1.30 | 1.16 | 1.21 |
| **Thickness (μm)** | | 14.0 | 13.8 | 15.2 | 10.7 | 9.8 | 9.4 | 9.4 |
| **Areal Capacitance (mF cm^-2^)** | **50 mV s^-1^** | 50.6 | 71.8 | 94.4 | 75.9 | 97.6 | 60.6 | 11.2 |
|  | **200 mV s^-1^** | 45.6 | 65.6 | 85.9 | 58.8 | 43.2 | 19.0 | 3.9 |
| **Volumetric Capacitance(F cm^-3^)** | **50 mV s^-1^** | 52 | 62.1 | 70.9 | 99.6 | 64.5 | 12.0 | 36.1 |
|  | **200 mV s^-1^** | 47.5 | 56.5 | 55.0 | 44.1 | 20.3 | 4.16 | 32.6 |

1. **Performance of rGO-PEDOT/PSS (33 wt. % GO) device**


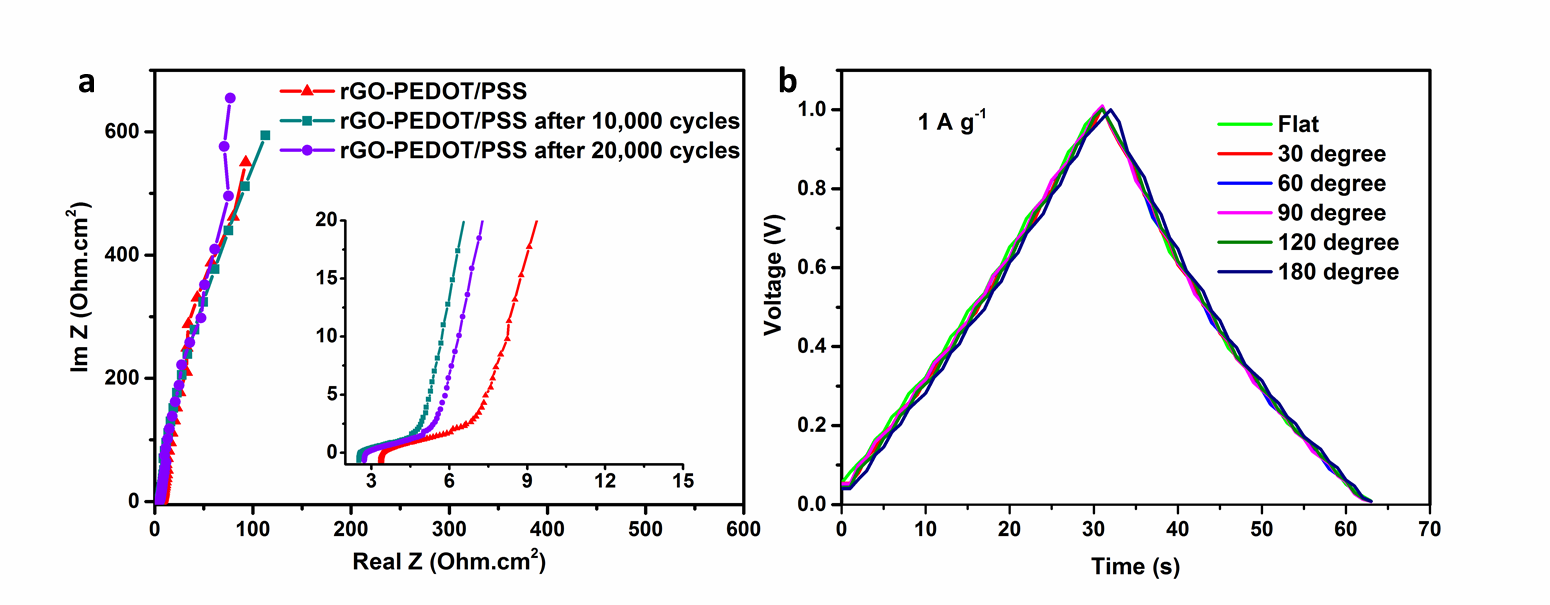


**Figure S9 |** (a) Nyquist Plot of rGO-PEDOT/PSS (33 wt. % GO) device prior to and after 10,000 and 20,000 cycles. (b) Charge/discharge curves of rGO-PEDOT/PSS (33 wt. % GO) device at different bendable state

1. **Higher mass loading**


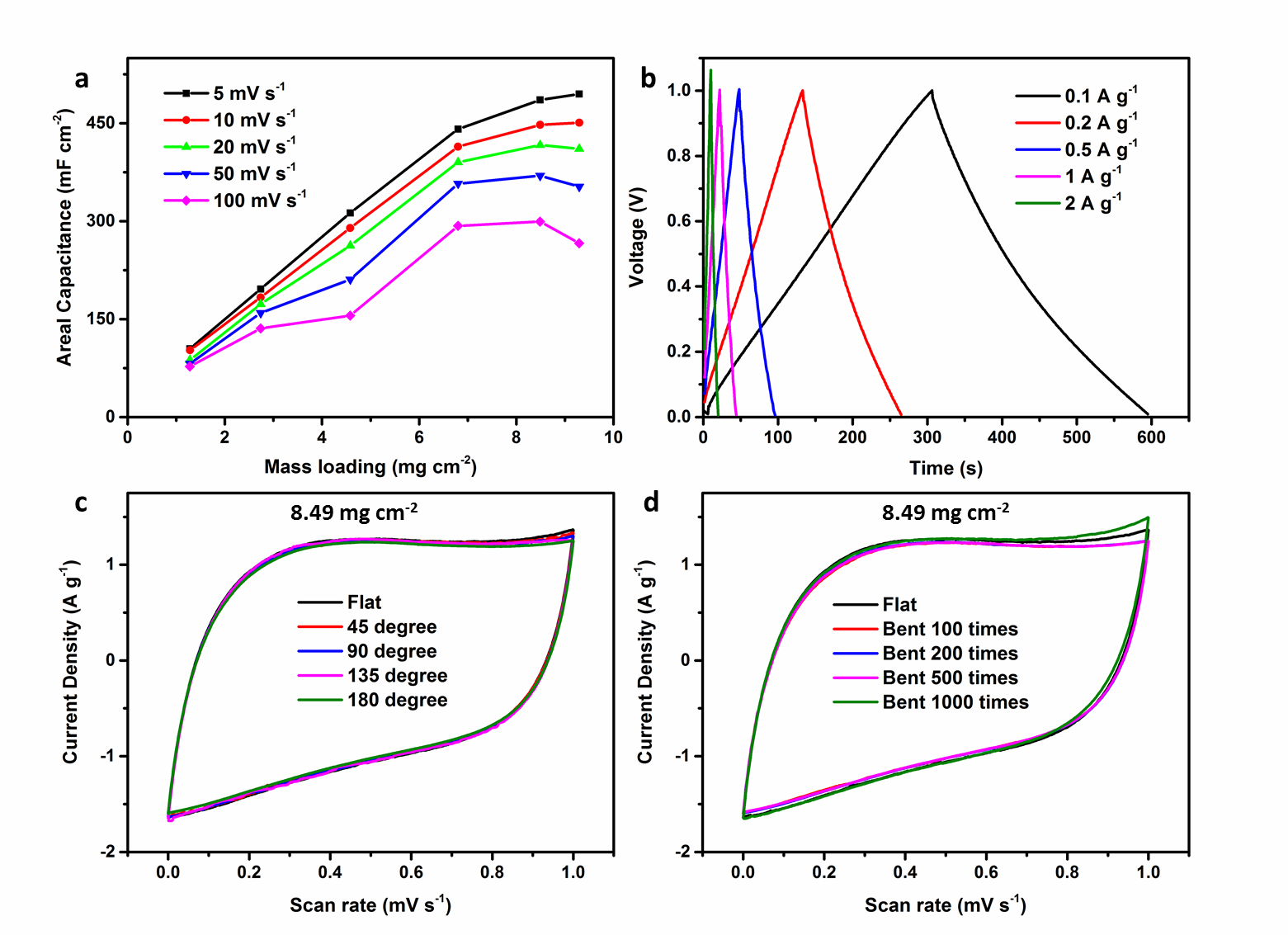


**Figure S10 | Electrochemical performance of rGO-PEDOT/PSS (33 wt. % GO) composite device with higher electrode mass loadings.** (a) Areal capacitance versus mass loading at different scan rates. (b) charge-discharge curves of composite device with a high mass loading of 8.49 mg cm^-2^. CVs of composite device with a mass loading of electrode 8.49 mg cm^-2^ at different bendable state (c) after bended different times (d) at a scan rate of 50 mV s^-1^

Table S2 Calculated specific capacitance of rGO in the composite film based on the mass of rGO and Equation S1

| Sample | Content of GO | Content of PEDOT/PSS | Capacitance of composite electrode (F g^-1^) (5 mV s^-1^) | Capacitance (F g^-1^) of rGO based on rGO |
| --- | --- | --- | --- | --- |
| 20% GO | 20% | 80% | 68.7 | 227.9 |
| 33% GO | 33% | 67% | 81.68 | 188.8 |
| 50% GO | 50% | 50% | 87.2 | 145.5 |
| 67% GO | 67% | 33% | 98.54 | 132.8 |
| 80% GO | 80% | 20% | 110.65 | 131.1 |
| rGO | 100% | 0% | 56.11 | 56.11 |

|  | $C_{g}\times P_{g}+C_{p}\times P_{p}=C$ | (S1) |
| --- | --- | --- |

Since most of the capacitance derives from rGO, gravimetric capacitances of rGO (C_g_) in the composite films were calculated according to Equation S1, where C and C_p_ is the gravimetric capacitance of the composite electrode and pristine PEDOT/PSS at 5 mV s^-1^ respectively, P_g_ and P_p_ is the mass percentage of GO and PEDOT/PSS in composite film. It can be seen that Cg in composite (33 wt. % GO loading ) is 189 F g^-1^ (Table S2), which is comparable with other literature reported values for both graphene and polymer based materials as list in Supplementary Table S3^4,5,8,10,12^, while the capacitance of pure rGO film is only 56.11 F g^-1^. This can be explained to be the result of effective prevention of rGO’s restacking problem by the addition of PEDOT/PSS. In addition, the higher loading of PEDOT/PSS in the composite resulted in higher C_g_, which indicated that high PEDOT/PSS loading can effectively prevent the re-stacking of rGO sheets.

**Table S3. The gravimetric, areal and volumetric capacitance of some flexible solid-state SCs (NA: not available)**

|  | Mass loading (mg cm^-2^) | Thickness (μm) | Electrolyte | Test condition | F g^-1^ | mF cm^-2^ | F cm^-3^ |
| --- | --- | --- | --- | --- | --- | --- | --- |
| PEDOT-paper ^4^ | 1 | 32 | PVA-H_2_SO_4_ | 0.4 A g^-1^ | 115 | 115 | 35 |
|  |  |  |  |  |  |  |  |
|  |  |  |  |  |  |  |  |
| Graphene hydrogel ^5^ | 2 | 120 | PVA-H_2_SO_4_ | 1 A g^-1^ | 186 | 372 | 31 |
| PANi-Au-paper ^6^ | 0.47 | NA | PVA-H_2_SO_4_ | 0.2A g^-1^ | ~212 | ~100 | NA |
| LSG ^7^ | 0.036 | 7.6 | PVA-H_3_PO_4_ | 4 A g^-1^ | NA | 7.34 | 9.66 |
| G-CNF Aerogel ^8^ | 1.52 | NA | PVA-H_2_SO_4_ | 5 mV s^-1^ | 207 | 316 | NA |
| CNT-BNC ^9^ | 0.4 | NA | Ion Gel | 1 A g^-1^ | 50.5 | 20.2 | NA |
| Graphene-cellulose paper ^10^ | 0.675 | NA | PVA-H_2_SO_4_ | NA | 136.3 | 92 | NA |
| CNT-PET ^11^ | NA | 20 | PVA-H_3_PO_4_ | NA | NA | <40 | 20 |
| Planar graphene ^12^ | 0.00158 | NA | PVA-H_3_PO_4_ | 0.281 uA cm^-2^ | 247 | 0.394 | NA |
| **This work** | 1.28 | 15.2 | PVA-H_3_PO_4_ | 10 mV s^-1^ | 79.9 | 102 | 67.3 |
|  | 8.49 | 89.7 |  |  | 52.7 | 448 | 49.9 |

***the value is calculated according to the data given in paper.**

**Calculations**

1. **Capacitance of device**

From CV

|  | 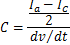 | (S2) |
| --- | --- | --- |

From charge-discharge

|  | 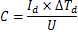 | (S3) |
| --- | --- | --- |

1. **Specific capacitance of single electrode (all the specific capacitances in the main text without specific note are based on the mass, area or volume of single electrode film)**

From CV

|  | 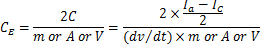 | (S4) |
| --- | --- | --- |

From charge-discharge

|  | 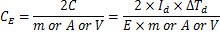 | (S5) |
| --- | --- | --- |

1. Specific capacitance of whole device

|  | 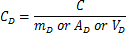 | (S6) |
| --- | --- | --- |

1. Energy density and Power density of supercapacitor device

|  | 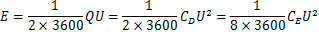 | (S7) |
| --- | --- | --- |
|  | 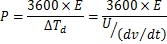 | (S8) |

C= capacitance of device (F)

C_E_=specific capacitance of single electrode (F g^-1^ or F cm^-2^ or F cm^-3^)

C_D_=specific capacitance of whole device (F g^-1^ or F cm^-2^ or F cm^-3^)

I_a_= anodic current (A) at 0.5V

I_b_= cathodic current (A) at 0.5V

dv/dt = scan rate (V s^-1^)

U=E_1_-E_2_= potential window (V)

I_d_ = Constant current used for charging and discharging(A)

ΔT_d_ = discharge time (s)

m = mass of single electrode (g)

A = area of single electrode (cm^2^)

V = volume of single electrode (cm^3^)

m_D_ = mass of whole device (g)

A_D_ = area of whole device (cm^2^)

V_D_ = volume of whole device (cm^3^)

E = energy density (Wh g^-1^ or Wh cm^-2^ or Wh cm^-3^)

P = power density (W g^-1^ or W cm^-2^ or W cm^-3^)

**References**

1 Stankovich, S. *et al.* Synthesis of graphene-based nanosheets via chemical reduction of exfoliated graphite oxide. *Carbon* **45**, 1558-1565 (2007).

2 Chen, J. *et al.* Scalable solid-template reduction for designed reduced graphene oxide architectures. *ACS Appl. Mater. Interfaces* **5**, 7676-7681 (2013)

3 Li, J., Xiao, G., Chen, C., Li, R. & Yan, D. Superior dispersions of reduced graphene oxide synthesized by using gallic acid as a reductant and stabilizer. *J. Mater. Chem. A* **1**, 1481-1487 (2013).

4 Anothumakkool, B., Bhange, S. N., Soni, R. & Kurungot, S. Novel scalable synthesis of highly conducting and robust PEDOT paper for high performance flexible solid-supercapacitor. *Energy Environ. Sci.* **8** 1339-1347 (2015).

5 Xu, Y. *et al.* Flexible solid-state supercapacitors based on three-dimensional. *ACS Nano* **7**(5), 4042-4049 (2013).

6 Yuan, L. *et al.* Paper-based supercapacitors for self-powered nanosystems. *Angew. Chem. Int. Ed.*  **51**, 4934-4938 (2012).

7 El-Kady, M. F., Strong, V., Dubin, S. & Kaner, R. B. Laser scribing of high-performance and flexible graphene-based electrochemical capacitors. *Science (New York, N.Y.)* **335**, 1326-1330 (2012).

8 Gao, K. *et al.* Cellulose nanofiber–graphene all solid-state flexible supercapacitors. *J. Mater. Chem. A* **1**, 63-63 (2013).

9 Kang, Y. J. *et al.* All-solid-state flexible supercapacitors fabricated with bacterial nanocellulose papers , carbon nanotubes , and triblock-copolymer ion gels. *ACS Nano* **6** (7), 6400-6406 (2012).

10 Weng, Z. *et al.* Graphene-cellulose paper flexible supercapacitors. *Adv. Energy Mater.*  **1**, 917-922 (2011).

11 Kaempgen, M., Chan, C. K., Ma, J., Cui, Y. & Gruner, G. Printable thin film supercapacitors using single-walled carbon nanotubes. *Nano lett.* **9**, 1872-1876 (2009).

12 Yoo, J. J. *et al.* Ultrathin planar graphene supercapacitors. *Nano lett.* **11**, 1423-1427 (2011).
